# The associations between playing a musical instrument and grey matter in older adults at risk for dementia: a whole-brain VBM analysis

**DOI:** 10.1007/s11357-025-01844-x

**Published:** 2025-09-03

**Authors:** Nicole Espinosa, Marshall A. Dalton, Hannes Almgren, Andrew C. McKinnon, Helen F. Mitchell, Zoe Menczel Schrire, Sharon L. Naismith

**Affiliations:** 1 School of Psychology, Faculty of Science, University of Sydney, Sydney, NSW Australia; 2 Healthy Brain Ageing Program, Brain and Mind Centre, University of Sydney, 94 Mallett Street, Camperdown, Sydney, NSW Australia; 3 School of Biomedical Engineering, Faculty of Engineering, University of Sydney, Sydney, NSW Australia; 4 Sydney Conservatorium of Music, University of Sydney, Sydney, NSW Australia; 5 School of Psychology, Western Sydney University, Sydney, NSW Australia; 6 Charles Perkins Centre, University of Sydney, Sydney, NSW Australia

**Keywords:** Imaging, Music, Aging, Dementia, Grey matter

## Abstract

While research suggests that playing musical instruments promotes neuroplasticity in professional musicians, it remains unclear whether lifelong music experience benefits brain health in non-professional musicians. This study examined whether playing a musical instrument across the lifespan is associated with (a) altered grey matter (GM) density and (b) neuropsychological functioning in older adults at risk for dementia. Sixty-one individuals aged ≥ 50 years were recruited from a memory clinic. Participants underwent magnetic resonance imaging and neuropsychological assessment from which composite scores for learning, memory, and executive functioning were derived. Based on musical history, participants were classified as: active players (*n* = 15), former players (*n* = 20), and naïve (*n* = 26). Voxel-based morphometry analyses, correcting for age and total intracranial volume, assessed GM density differences. General linear models, controlling for age, tested associations between music experience and cognition. Active players showed increased GM density in the left planum temporale (*p* < 0.0001), left planum polare (*p* < 0.0001), right posterior insula (*p* < 0.0001), and left cerebellum exterior (*p* < 0.0001) compared to the naïve group. They also showed increased GM in the left cerebellum exterior (*p* < 0.0001) relative to former players. No GM differences were observed between former players and naïve individuals. Music experience was not significantly associated with neuropsychological performance. In older adults at risk for dementia, currently playing a musical instrument was associated with increased GM density in regions linked with musical training. Further research is needed to explore music’s role in brain health and dementia prevention.

**Graphical Abstract:**

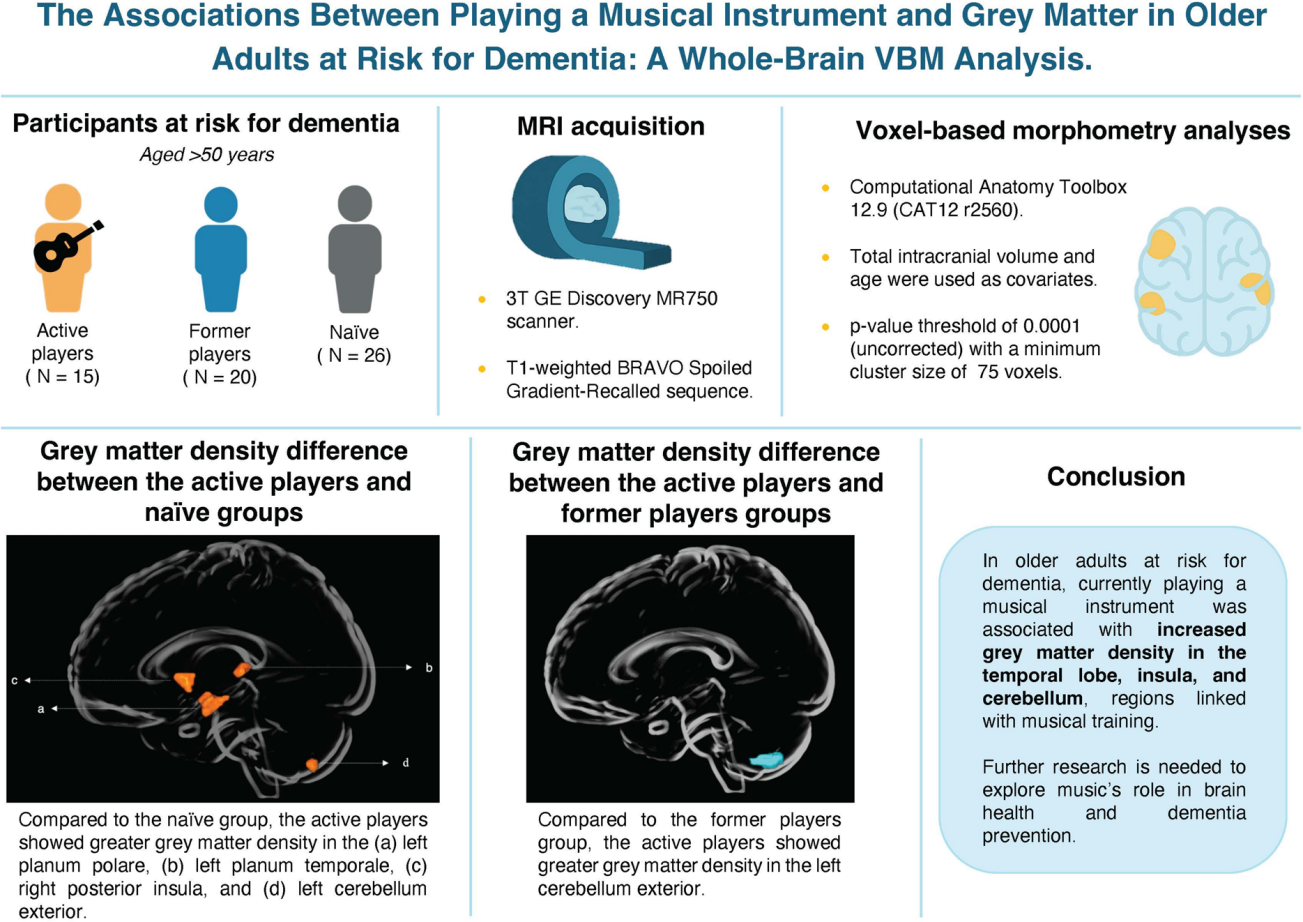

**Supplementary Information:**

The online version contains supplementary material available at 10.1007/s11357-025-01844-x.

## Introduction

As individuals age, they often exhibit diverse cognitive trajectories. While some maintain intact cognitive abilities, others might experience subjective and/or objective cognitive decline [1–3]. Studies have shown that individuals with subjective cognitive decline (SCD) (i.e. without objective impairment on testing) are twice as likely to develop dementia compared to those without such complaints [4]. Similarly, older adults with mild cognitive impairment (MCI), characterised by objective cognitive decline but intact daily functioning [5], are at a high risk of progressing to dementia, with approximately 45% converting to dementia within five years [5]. Both SCD and MCI may be a preclinical period of dementia, during which amyloid, and potentially other (e.g. cerebrovascular, alpha synuclein) pathology begins to accumulate in the brain [6]. Importantly, in addition to amyloid burden, evidence indicates that the decline in cognitive function observed in this period, to some extent, is mediated by structural and functional brain changes [7–9], including hippocampal atrophy, cortical thinning in the frontal and parietal lobes, white matter lesions, and reduced white matter integrity and functional connectivity in the default mode network [9–13]. Since 45% of dementia risk is associated with modifiable risk factors [14], recognising and addressing these early changes in brain integrity and cognition are key for dementia prevention efforts.

Cognitive activity is considered to be a modifiable dementia risk factor [14]. Engaging in enriching activities throughout life, such as learning new skills, social engagement, and cognitive training, may promote cognitive reserve and neuroplasticity. Cognitive reserve enhances the brain’s resilience to neuropathological damage, helping it to compensate for age-related changes and pathology [15, 16], whilst neuroplasticity refers to the brain’s ability to adapt to changing environmental conditions by forming new neural connections, supporting the development of cognitive reserve [17].

Music engagement may be beneficial for cognitive reserve and neuroplasticity, in turn offering protection against brain neurodegeneration and cognitive decline [18, 19]. A systematic review and meta-analysis found that lifetime musical experience was associated with a lower incidence of cognitive impairment and dementia [20]. Additionally, a cohort study by Vetere et al. [21] found small but clinically meaningful effect sizes for better performance in working memory (*d* = 0.24) and executive functioning (*d* = 0.17) in older adults with (vs. without) a history of playing a musical instrument. While this preliminary body of work supports the notion that lifelong engagement in musical activities may be associated with enhanced cognitive performance, the mechanisms underpinning this association are unknown.

Some mechanistic insights can be gleaned from prior work focused on younger adults with different levels of musical expertise. This work has shown that extensive musical practice (i.e. 10 or more hours of practice per week) promoted structural and functional changes predominantly in the auditory-motor network, which is closely linked to the temporal, cerebellar, and parietal regions [22, 23]. Other brain regions found to differ structurally between musicians and non-musicians include those that support memory, working memory and language, such as the hippocampus [24] and the prefrontal cortex [25, 26]. Critically, these differences appear to be moderated by hours of music practice, music proficiency, age at which music training started [22, 26] and the type of musical instrument played (e.g. piano or woodwind instruments) [27]. Further, a meta-analysis of 48 cross-sectional studies examining grey matter volumes and functional brain differences between musicians and non-musicians revealed significant group differences across several brain regions. These include the superior temporal gyrus, Heschl’s gyrus, planum temporale, insula, frontal gyrus, precentral gyrus, cerebellum, and premotor cortex [28]. Cross-sectional studies in healthy younger adult musicians, therefore, suggest music playing is linked with structural and functional integrity in the auditory-motor cortex.

For non-professional musicians, it remains unclear if playing a musical instrument confers benefits and whether these benefits of musical instrument playing may need to be maintained throughout the lifespan. Two studies of predominantly healthy adults revealed that older adults with more music experience had greater grey matter (GM) volumes in the parahippocampus and inferior frontal cortex [29], greater functional connectivity between the insula and the prefrontal cortex, postcentral and precentral gyrus, and decreased functional connectivity between the ventral anterior insula and thalamus [30]. However, a notable limitation of these studies is that the musical self-report data was collected 5 to 8 years after the MRI data, creating a temporal mismatch which complicates the ability to establish clear causal links between musical activity and structural brain changes. To date, no known study has investigated the impact of lifetime music experience on neuroplasticity and cognition in older adults at risk for dementia, such as in those with SCD or MCI. Given that this represents a critical window for secondary prevention in people already experiencing cognitive symptoms, it is crucial to determine whether playing a musical instrument later in life is associated with superior brain integrity. This knowledge, in turn, could inform the design and timing of musical interventions for mitigating cognitive decline.

The primary aim of this study was to investigate in older adults at risk for dementia how a lifetime history of playing a musical instrument was associated with GM density using a data-driven approach. Specifically, we sought to ascertain whether continued lifetime vs. prior music engagement differs from being musically naïve in terms of brain integrity. The secondary aim was to examine any relationship with current neuropsychological functioning across the domains of learning, memory, and executive functioning. It was hypothesised that current music playing would confer benefits in regions previously shown to be linked to music engagement (i.e. auditory-motor areas) compared to those who ceased practising music and those without any lifetime music experience. We also hypothesised that current players would demonstrate superior memory, learning, and executive functioning.

## Methods

### Participants

Participants aged ≥ 50 years at risk for dementia were recruited from the Healthy Brain Ageing (HBA) clinic at the Brain and Mind Centre, University of Sydney. The clinic receives referrals from general practitioners for individuals with new (i.e. within the last five years) cognitive concerns. Exclusion criteria are Mini-Mental State Examination (MMSE) [31] score < 24; significant neurological disorder (e.g. epilepsy, Parkinson’s disease), previous stroke or head injury with loss of consciousness exceeding 30 min; intellectual disability; history of significant psychiatric illness other than major depression (e.g. bipolar disorder, schizophrenia); and current or past substance misuse or abuse. To be included in this study, participants were also required to have completed the ‘Music for Neuroplasticity Questionnaire’ as well as MRI scanning (see below).

All participants provided written informed consent before participation in the study, and all research activities were approved by the University of Sydney Human Research Ethics Committee (Protocol number: 2012/1873).

### Medical and mental health assessment

Medical history, medication use, and alcohol consumption were recorded by a geriatrician. Medical burden was rated using the Cumulative Illness Rating Scale (geriatric version) (CIRS-G) [32], and height and weight information were measured to determine body mass index (BMI). The MMSE was administered to participants to describe global cognition. A research psychologist completed the Mini International Neuropsychiatric Interview (MINI), to determine history of common psychiatric disorders [33]. Depressive symptoms over the prior week were self-reported using the 15-item Geriatric Depression Scale (GDS-15) [34].

### Neuropsychological assessment

A clinical neuropsychologist administered a comprehensive standardised test battery, which was designed to assess aspects of cognition relevant to ageing and dementia. For this study, we were specifically interested in tests that have been shown to be relevant for ageing and music experience [21]. Hence, we included the following composite scores:

1. Verbal learning composite: the Logical Memory I subtest of the Wechsler Memory Scale-III (WMS-III) [35] and the Rey Auditory Verbal Learning Test (RAVLT) trials 1–5 total score [36].
2. Verbal memory composite: the Logical Memory II subtest of the WMS-III [35] and the RAVLT trial 7 score [36].
3. Executive functioning composite: the Delis–Kaplan Executive Functioning System Color Word Interference Test conditions 3 and 4 [37], the Trail Making Test (TMT) part B [38], and the Controlled Oral Word Association Test (COWAT, letters F, A, S) [39].

Cognitive composites were constructed following the methodology outlined in our previous work [40]. Briefly, first, we computed the age-adjusted standard *z*-scores for each individual test. Next, an average *z* score was determined for each participant by calculating the mean of all test scores within a given composite, ensuring that each test contributed equally (e.g. verbal learning composite: ((RAVLT trial 7 *z* score + Logical Memory II *z* score)/2). For descriptive purposes, the Wechsler Test of Adult Reading (WTAR) [41] is reported as an estimate of premorbid intellectual functioning.

As described previously [42], participants’ cognitive status was rated via consensus of two neuropsychologists and a geriatrician for classification of SCD only or MCI [43]. MCI classification required generally intact activities of daily living, yet evidence of ≥ 1.5 standard deviation (SD) decline on neuropsychological testing relative to age and level of education. Based on established criteria [44], participants were also classified as having amnestic MCI (aMCI) or non-amnestic (naMCI) MCI depending on the presence or absence of deficits in verbal memory recall, respectively [5] and as single or multiple domain MCI.

### Music for neuroplasticity questionnaire

This is a 23-item self-report questionnaire measuring lifetime music experience that was developed by researchers from the HBA program (SN, NE). It consists of three sections: (1) lifetime instrumental playing experience, (2) lifetime singing experience, and (3) interest in learning music. The aim was to capture a variety of musical behaviours, experiences, and preferences. For this cross-sectional study, we analysed data exclusively from Sect. 1 of the questionnaire, which focused on lifetime instrumental playing. The following information was collected:

- Lifetime experience with playing an instrument.
- Age at which they started playing an instrument.
- Instruments that they used to play or that they are currently playing.
- Years of music practice.
- Hours of music practice per week.
- Years since they stopped playing an instrument.

Based on this information, participants were classified into three groups: (a) currently playing an instrument (i.e. active players), (b) history of playing an instrument (i.e. former players), and (c) no instrument-playing experience (i.e. naïve). Those in the former players group had more than 5 years of music experience but stopped playing more than 10 years ago. The criterion for years of musical experience was selected based on previous studies that have consistently defined amateur musicians as those who have engaged in regular musical practice for at least 6 years [45]. A cut-off of more than 10 years since cessation was chosen to allow sufficient time for potential long-term structural effects of music training to stabilise or diminish. The Music for Neuroplasticity Questionnaire is provided in supplementary document 1.

### Magnetic resonance imaging acquisition

Participants completed the MRI protocol within 3 months of the clinical assessment at the Brain and Mind Centre, Sydney, Australia, using a 3-Tesla General Electric (GE) Discovery MR750 scanner (GE Medical Systems, USA) with a 32-channel phased-array head coil. For this study, the following T1 weighted imaging sequence was used: 3D-T1-weighted BRAVO spoiled gradient-recalled (SPGR) (repetition time = 7.4 ms; echo time = 3.0 ms; flip angle = 11; matrix 256 × 256; 1.0 mm isotropic voxels).

### Structural MRI data pre-processing

For whole-brain GM analysis, the structural MRI data underwent processing and analysis using the Computational Anatomy Toolbox 12.9 (CAT12 r2560) [46] integrated into SPM12 (Statistical Parametric Mapping software; http://www.fil.ion.ucl.ac.uk/spm/software/spm12/). This analysis was carried out on MATLAB R2023a (The MathWorks, Natick, MA, USA).

For the VBM data pre-processing, we followed a standardised protocol with pre-defined parameters, as specified in the CAT12 Online Manual (https://neuro-jena.github.io/cat12-help/#module1). Briefly, this protocol involved several steps: (1) TI images were spatially registered to the tissue probability maps and then segmented into white matter, grey matter (GM), and cerebrospinal fluid. The voxel size for normalised images was 1.5 mm isotropic, and the corresponding strength of skull-stripping was 0.50. (2) After pre-processing, the quality of each image was assessed by visual inspection, the weighted overall image quality (IQR) index, and quartic mean *z* score obtained from the “check homogeneity” function in CAT12. Images with artefacts and a IQR less than C + were excluded. (3) GM images were smoothed with an 8-mm full-width at half-maximum Gaussian kernel. (4) Total intracranial volume (TIV) was estimated by CAT12 as a covariate to correct for different brain sizes between participants.

### Statistical analysis

Data analysis was performed using the Statistical Package for Social Sciences (SPSS version 28, Armonk, NY: IBM Corp.). Differences in demographic and clinical data between active players, former players, and naïve groups were assessed using one-way ANOVA, Kruskal–Wallis tests and Mann–Whitney *U* for non-normally distributed variables or chi-square (*X*^2^) tests for categorical variables. General linear models (GLMs), controlling for age, were conducted to examine differences in memory, learning and executive functioning among the three music groups. All analyses were two-tailed, with the alpha-level set to 0.05.

The statistical analysis for VBM data processing was performed with CAT12 statistical module. The smoothed data were used to perform an ANCOVA to identify significant GM volume differences between our three groups. TIV and age were used as covariates (nuisance variables) in the VBM analyses to identify potential effects of these variables. Because of the relatively small sample size, our statistical power would have been very low using family-wise error rate (FWER) or false discovery rate (FDR) corrections. Therefore, we applied a strict significance threshold of 0.0001 (uncorrected) with a minimum cluster size of 75 voxels.

As a sensitivity analysis and to control for any effect of age at which participants began learning music, we re-ran the VBM analysis excluding subjects who began learning music after the age of 50 to ensure that the comparison between groups reflects a more typical trajectory of musical training (*n* = 4, all part of the active players group), since learning music later in life may involve different cognitive and neural processes compared to those developed during earlier stages of life [47, 48]. In order to account for the effect of different musical instruments played by participants on brain structure, we conducted an exploratory VBM analysis, focusing exclusively on the active players and former players groups. We included the types of musical instruments that were significantly different between groups (i.e. piano and string instruments) as covariates in this analysis. Finally, to ensure sex differences did not influence our findings, we reran the main VBM analysis controlling for age, sex, and TIV. Given the limited sample size, sex was not included in the main analysis but evaluated separately in a sensitivity analysis.

## Results

### Participant characteristics

An initial 65 participants were recruited for the study. Of these, four cases were excluded due to poor MRI scan quality. Table 1 shows the demographic and clinical variables for the final sample of 61 individuals (mean age = 69.8, females = 62.3%, mean MMSE = 28.9, mean WTAR IQ = 106.4). Of this sample, 39.3% (24/61) met clinical criteria for MCI. Among these, 75% (18/24) were classified as having amnestic MCI, and 79.2% (19/24) had multiple-domain MCI. Ninety-five per cent (58/61) reported right-hand dominance. The sample included 15 active players, 20 former players, and 26 naïve participants.

**Table 1:** Demographic and clinical data for the three groups: currently playing instruments (active players), history of playing instruments (former players) or those whom have never played a musical instrument (naïve)

Measure	Active players			Former players			Naïve			df	*p* value
	*N*	Mean (Md)	SD (IQR)	*N*	Mean (Md)	SD (IQR)	*N*	Mean (Md)	SD (IQR)		
Age (years)	15	69.21 (70.35)	8.17	20	69.10 (66.67)	9.13	26	70.61 (70.83)	6.66	60	0.776
Sex: % female (*n*/*N*)	15	53.33	(8/15)	20	70.00	(14/20)	26	61.16	(16/26)	2	0.599
Education (years)	15	14.80 (15.00)	3.08	20	16.05 (16.00)	2.28	26	14.50 (15.00)	3.00	60	0.169
MCI classification: % (*n*/*N*)	15	40.00	(6/15)	20	35.00	(7/20)	26	42.31	(11/26)	2	0.880
MCI subtypes: % amnestic MCI (*n*/*N*)	6	66.67	(4/6)	7	100.00	(7/7)	11	63.64	(7/11)	2	0.191
MCI domains: % multiple MCI (*n*/*N*)	6	83.33	(5/6)	7	85.71	(6/7)	11	72.72	(8/11)	2	0.770
Cognition (MMSE)*	15	29.20 (29.00)	0.94 (1.00)	20	28.95 (29.00)	1.39 (2.00)	25	28.56 (29.00)	1.78 (3.00)	59	0.747
Right-handedness: % (*n*/*N*)	15	100.00	(15/15)	20	100.00	(20/20)	26	88.46	(23/26)	2	0.374
Antidepressant use: % (*n*/*N*)	15	25.00	(4/16)	19	20.00	(4/20)	25	28.00	(7/25)	2	0.865
Depressive symptoms (GDS-15)*	15	2.80 (2.00)	3.88 (3.00)	20	2.55 (2.00)	2.63 (2.00)	26	3.46 (1.00)	4.20 (8.25)	60	0.967
Body mass index*	15	27.72 (26.00)	6.37 (7.30)	18	25.87 (24.85)	6.35 (6.33)	23	26.94 (24.70)	5.17 (6.40)	55	0.439
Medical burden (CIRS-G)	15	7.27 (7.00)	2.15	19	6.68 (6.00)	4.77	25	6.92 (7.00)	2.89	58	0.889
Alcohol, units per week*	14	4.39 (2.25)	6.44 (7.25)	18	4.03 (1.50)	4.51 (8.25)	25	4.46 (3.00)	4.47 (7.00)	56	0.894
Age at which began learning music (years)^+^	15	28.53 (15.00)	26.98 (60.00)	20	9.20 (9.50)	2.24 (2.75)	-	-	-	35	**0.011**
Years of music practice^+^	15	9.87 (4.00)	13.03 (8.00)	20	8.90 (7.50)	3.65 (3.75)	-	-	-	35	0.053

*MCI* = Mild cognitive impairment; *MMSE* =Mini-Mental State Examination; *CIRS-G* = Cumulative Illness Rating Scale-Geriatric; *GDS-15* = 15-item Geriatric Depression Scale; *SD* = standard deviation; *Md *= Media; *IQR* = Interquartile range

All test statistics were one-way ANOVA analyses unless otherwise specified. Chi-square test was used for categorical data: antidepressant use, gender and MCI diagnosis, subtypes, and domains. All missing data were excluded using pairwise deletion. Bold values represent significant differences *p* < 0.05

*Kruskal-Wallis H test; +Mann-Whitney U test

There were no significant group differences with regards to age, sex, education, proportion with MCI or MCI subtypes (i.e. amnestic and non-amnestic, single or multiple domain), global cognition (MMSE), BMI, medical burden (CIRS-G), handedness, alcohol consumption, current depressive symptoms (GDS-15), or antidepressant use (Table 1).

For the two groups with music experience, none of the participants were professional musicians; all participants were amateur musicians. The active players group started learning music at a significantly later age (mean age = 28.5, standard deviation [SD] = 26.9, range = 4–75) when compared to the former players group (mean age = 9.2, SD = 2.2, range = 5–14; *p* = 0.011). While there was a trend for active players to have more years of music practice (9.9 years) than former players (8.9 years), this result was marginally non-significant (Table 1).

A significant difference was observed between the active players and former players in terms of the types of instruments they played, with significant differences between groups regarding piano (*p* < 0.001) and string instruments (*p* < 0.001) (Table 2). Specifically, 20% of active players and 80% of former players reported playing the piano. Playing string instruments (e.g. guitar or ukulele) was noted in 67% of active players, compared to only 10% of former players. Additionally, 20% of active players and 50% of former players played woodwind instruments (e.g. flute, recorder or clarinet). Active players reported practising approximately 1.93 h per week (SD = 1.14, range = 1–4). All participants from the former players group stopped practising music 10 or more years ago. The most common reasons cited for stopping playing an instrument among the former players were lack of time (45%), loss of interest (25%), and perceived lack of musical ability (10%).

**Table 2:** Distribution of musical instrument types by group

Group	Piano			String			Woodwind		
	*N* (%)	df	*p* value	*N* (%)	df	*p* value	*N* (%)	df	*p* value
Active players	3 (20%)	1	** < 0.001**	10 (67%)	1	** < 0.001**	3 (20%)	1	0.069
Former players	16 (80%)			2 (10%)			10 (50%)		

Active player = Currently playing an instrument; Former players = More than 5 years of music experience but stopped playing more than 10 years ago. Chi-square test was used for this analysis. Bold values represent significant differences p < 0.05.

### Group differences using VBM analysis

**Fig. 1:**
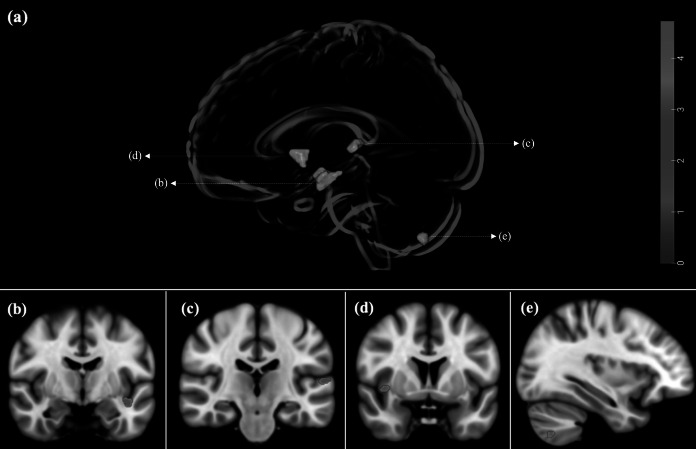
Grey matter density difference between those who currently play a musical instrument and those without any music experience. **a** Brain regions showing greater grey matter (GM) density in those who currently play an instrument as compared to those without music experience. Compared to those without music experience, those who continued playing an instrument showed greater GM density in the **b** left planum polare, **c** left planum temporale, **d** right posterior insula, and **e** left cerebellum exterior. Note: Age and TIV were included as covariates in the model. Analyses are reported at *p* < 0.0001, uncorrected for multiple comparisons with a minimum cluster size of 75 voxels. Colours represent *t* values

**Fig. 2:**
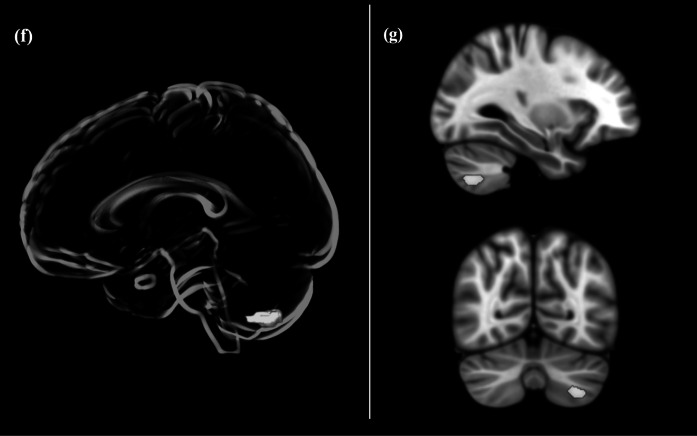
Grey matter density difference between those who currently play a musical instrument and those who used to play music but stopped more than 10 years ago. (f) Brain regions showing greater grey matter (GM) density in those who currently play an instrument as compared to those who used to play music but stopped. Compared to those who used to play music but stopped, those who continued playing an instrument showed greater GM density in the (g) left cerebellum exterior. Note: Age and TIV were included as covariates in the model. Analyses are reported at *p* < 0.0001, uncorrected for multiple comparisons with a minimum cluster size of 75 voxels. Colours represent *t* values

The active players group showed increased GM density in the left planum temporale, left planum polare, right posterior insula, and left cerebellum exterior, when compared to the naïve group (Table 3 and Fig. 1). Further, compared to former players group, active players group showed increased GM density in the left cerebellum exterior (Table 3 and Fig. 2). For subject-specific estimates for each group, see supplementary Figs. 1 and 2. No significant differences in GM density were found between the former players and naïve groups. When sex was included as a covariate, the significant group differences remained unchanged.

**Table 3:** Brain regions with increased grey matter density in older adults who currently play a musical instrument

Structure	Number of voxels	Peak MNI coordinate			*p* value	*T* value
		*x*	*y*	*z*		
Contrast 1*						
Left planum temporale	119	− 62	−21	8	** < 0.0001**	4.95
Left planum polare	279	− 39	− 6	− 14	** < 0.0001**	4.83
Right posterior insula	191	42	0	− 3	** < 0.0001**	4.56
Left cerebellum exterior	78	− 33	− 66	− 50	** < 0.0001**	4.33
Contrast 2*						
Left cerebellum exterior	300	− 30	− 68	− 48	** < 0.0001**	4.98

*Contrasts: Older adults who currently play a musical instrument compared to: (1) older adults without music experience; (2) older adults who have more than 5 years of music experience but stopped playing more than 10 years ago

Analyses are reported at *p* < 0.0001, uncorrected for multiple comparisons with a minimum cluster size of 75 voxels. Age and total intracranial volume were included as covariates in the analysis

Bold values represent significant differences *p* < 0.0001

### VBM sensitivity analysis

For the sensitivity analysis, we re-ran the VBM analysis excluding participants who began learning music after the age of 50. No significant differences remained between active and former players in the age at which they began learning music or years of music practice (Supplementary Table 1). When comparing the active players to the naïve group, several brain regions previously identified remained significant, including the left planum temporale, left planum polare, and right posterior insula. Additionally, new brain regions emerged as significantly different in this analysis. The active players exhibited greater GM density in the left and right precuneus, left and right subcallosal area, and the right inferior temporal gyrus compared to the naïve group. The left cerebellum exterior, which was significant in previous analyses, was not significant in this analysis (Supplementary Table 2 and Supplementary Fig. 3).

When comparing the active players to the former players group, the left cerebellum exterior continued to show significant differences. Additionally, increased GM density was observed in the right and left precuneus in the active players group when compared to the former players (Supplementary Table 2 and Supplementary Fig. 4). No significant differences in GM volumes were found between the former players and naïve groups.

### VBM group differences controlling for type of musical instruments

As an exploratory analysis and to control for the potential effects of playing different types of musical instruments on brain structure, those musical instruments that were significant between groups (i.e. piano and strings) were included as separate covariates in the VBM analysis. As in previous analyses, a significant result was observed in the left cerebellum (*p* < 0.0001, *t* = 5.28).

### Group differences in neuropsychological domains

A GLM analysis was conducted to explore the relationship between music experience and neuropsychological performance. The analysis revealed no significant group differences in verbal learning, verbal memory, and executive functioning composites (Table 4). These findings did not change when a sensitivity analysis was conducted excluding participants who began learning music after the age of 50 (*n* = 4) (Supplementary Table 3).

**Table 4:** Associations between neuropsychological performance and music experience

Measure	Active Players			Former Players			Naïve			Wald X^2^	η²ₚ	p-value
	N	Mean (Md)	IQR	N	Mean (Md)	IQR	N	Mean (Md)	IQR			
Verbal memory composite	15	0.41 (1.07)	2.12	19	0.39 (0.65)	1.73	26	0.24 (0.55)	1.65	0.17	0.003	0.919
Verbal learning composite	15	0.14 (0.71)	2.02	19	0.53 (0.61)	1.14	26	0.01 (0.08)	1.58	2.71	0.043	0.257
Executive functioning composite	15	0.66 (0.51)	0.45	19	0.51 (0.49)	0.70	26	0.28 (0.34)	0.66	4.59	0.064	0.100

Active player = Currently playing an instrument; Former players = More than 5 years of music experience but stopped playing more than 10 years ago; *Naïve =* No instrument-playing experience; *Md =* Media; *IQR* =  Interquartile range; *η*^2^*ₚ* = Partial eta squared

All test statistics were general linear models, controlling for age

Bold values represent significant differences *p* < 0.05

## Discussion

This is the first known study to investigate the association between lifetime history of playing a musical instrument on GM density and cognition in older adults at risk for dementia. Our findings revealed that currently playing of a musical instrument is associated with increased GM density in the temporal lobe, insula, and cerebellum compared to those who have never played or those who have stopped playing more than 10 years ago (despite playing for more than 5 years). Specifically, using a data-driven neuroimaging approach, we found increased GM density in the left planum temporale, left planum polare, left cerebellum exterior, and right posterior insula in the active players group compared to the naïve group. The active players group also show increased GM density in the left cerebellum exterior compared to the former players, even after controlling for types of musical instruments (i.e. piano and strings). No significant differences were found between the former players and naïve groups. There were no differences in neuropsychological performance between groups, with effect sizes ranging from very small to moderate (partial *η*^2^ = 0.003–0.064), indicating minimal group-level variance across neuropsychological outcomes.

The impact of music on the brain has been studied extensively, particularly as a marker of neuroplasticity in professional musicians. Notably, this study aligns with the aforementioned meta-analysis of younger professional musicians, which found that music practice is strongly associated with increased GM volumes in brain regions such as the planum temporale, insula, and cerebellum (28). Our study builds on this foundation, providing further evidence about the association between music training and increased GM density, specifically in the temporal lobe, insula and cerebellum, in older at risk for dementia. These findings underscore the need for further investigation into music training as a potential strategy to support neuroplasticity and brain health in this vulnerable population.

The observed association between actively playing music and GM density in the temporal lobe are aligned with the known role that the temporal lobe plays in music processing and the perception of higher-order musical features [49, 50]. The regions included the insula, the planum temporale, and the planum polare, which are critically involved in processing and analysing the complex sound features that support musical perception and cognition [49–51]. Importantly, these neuroanatomical differences were found to diminish in individuals who had ceased playing a musical instrument more than 10 years ago, suggesting that continuous musical practice may be necessary to sustain these structural brain changes and their related neuroplastic benefits.

In this study, the finding that active players had increased GM density in the left cerebellum exterior compared to former players and naïve groups is also consistent with the documented role of the left cerebellum in the processing of singing [52, 53] and movement timing accuracy [54, 55], both of which are essential for the fine motor coordination and precision required during music playing. Notably, unlike the temporal lobe findings, the cerebellar finding was apparent in the active player group when compared to individuals who discontinued playing an instrument. Whilst speculative, this may reflect the cerebellum’s central role in motor control and coordination, which are crucial for musical performance. The temporal lobe is primarily responsible for processing auditory information, including music listening, and higher-order musical processing [56]. In contrast, the cerebellum’s activation is closely tied to the practice of motor skills, which is typically enhanced through music playing. This distinction underscores the significance of sustained practice in preserving the motor benefits associated with music playing.

Our sensitivity analyses confirmed that the effects of currently playing a musical instrument persisted after excluding participants who began learning after the age of 50. We also identified increased GM density in the bilateral precuneus, subcallosal area, and inferior temporal gyrus in active players compared to other groups. These regions are associated with working memory processes [57], emotional regulation [58, 59], and the visual processing of musical notation [60, 61], respectively. Notably, the precuneus has been linked to early metabolic and structural changes in Alzheimer’s disease [62], suggesting that engaging in musical practice may support brain resilience in vulnerable areas. Similarly, the subcallosal area’s role in emotional regulation aligns with theories that sustained musical engagement fosters structural adaptations in emotion-related networks [58, 59]. While these findings align with previous research on music-related neuroplasticity, further studies are needed to determine their clinical significance and long-term impact on brain health.

In this study, the level of music engagement was not related to current neuropsychological performance in the domains of memory, learning, and executive functioning, which are implicated early in neurodegenerative disease and pivotal to daily functions. It is possible that the brain benefits of music are limited to regions such as the temporal cortex, insula, and cerebellum and may not extend to the hippocampus and prefrontal cortex areas that underpin these critical cognitive functions. It is however acknowledged from prior work that music practice can enhance cognition across the lifespan [63–65], and that current and past musicians have better attention, working memory, and executive functioning, compared to non-musicians [21, 66]. The absence of associated cognitive effects in our study may indicate that alterations in GM precede observable cognitive improvements [6], such that early neuroplastic adaptations may not yet have translated into measurable cognitive benefits. Alternatively, it is possible that in early preclinical or prodromal neurodegenerative stages, improving cognition via music making may be inherently challenging. Another possibility is that the study lacked sufficient power to detect small differences in cognitive performance. Perhaps other tests that probe auditory and motor function would reveal a tighter relationship between music engagement and these more closely linked aspects of cognition. Further research is needed to explore the relationship between music, neuroplasticity and neuropsychological functioning in older adults at risk for dementia.

This study has several limitations that warrant consideration. First, this is a cross-sectional study; hence, the time course of GM alterations with respect to music playing cannot be confirmed. Longitudinal studies are necessary to investigate the impact of playing a musical instrument on GM density throughout the lifespan. Second, in our VBM analysis, we applied a strict *p* value threshold and minimum cluster size to mitigate the risk of type I error. However, uncorrected analyses are still more vulnerable to detecting false findings compared to more rigorous correction methods, such FWER or FDR adjustments. Therefore, replication in larger samples with rigorous multiple comparison corrections is essential to strengthen the validity and reproducibility of these results. Third, the modest sample size, particularly the small number of current players (*n* = 15), limits the statistical power and generalisability of our findings. As such, the results should be considered preliminary and interpreted with caution. Replication in larger cohorts is essential to validate the observed group differences and enable more robust subgroup analyses. Fourth, music experience was assessed via a self-report questionnaire, which may be subject to recall bias. This is especially relevant given the ageing nature of the sample and the potential for memory inaccuracies among participants with cognitive impairment. Hence, prospective studies are warranted to build a clearer picture of the effect of music playing on GM density and cognition. Fifth, this study did not examine individuals who had ceased music training less than 10 years ago, limiting our ability to characterise more recent post-training effects. Future studies should investigate shorter cessation periods (e.g. 1–5 or 5–10 years) to better understand the timeline over which structural brain changes related to musical training may persist or diminish following discontinuation. Sixth, we acknowledge the limitation in our exploratory analysis due to insufficient power to control for the type of instruments in our model. Consequently, these results should be interpreted with caution. We recommend that future studies with larger sample sizes be conducted to further examine the effect of different musical instruments on neuroplasticity. Seventh, participants in our sample were highly educated and likely already had a degree of cognitive reserve. Our findings may not extend to the broader population, especially those with lower educational attainment. Finally, it is noted that the underlying pathophysiological mechanisms giving rise to cognitive concerns in our sample cannot be ascertained without the use of Alzheimer’s disease biomarkers [67], and therefore, the SCD and MCI samples are likely to have biological heterogeneity, which in turn, may have influenced our findings within and across groups. Future studies may wish to biologically characterise SCD and MCI participants.

The current study provides novel evidence of the association between actively playing a musical instrument and GM density in older adults at risk for dementia. Notably, the differences in GM density in the temporal lobe, insula, and cerebellum were present in the active players group compared to former players and naïve groups, which could mean that the benefits of music practice on brain structure are maintained through sustained and consistent engagement. Although we did not find significant associations between cognitive performance and music experience, the observed structural differences may indicate a potential protective effect against regional neurodegeneration in these areas. Thus, our findings underscore the importance of regular musical activity as a potential non-pharmacological approach to supporting brain health in aging populations. Further studies with larger sample sizes, longitudinal designs, and robust randomised controlled trials in people at risk for dementia are needed to confirm the potential benefits of music playing on neuroplasticity, cognitive resilience, and dementia prevention.

## Supplementary Information

Below is the link to the electronic supplementary material.

MOESM 1 (DOCX 1,155 KB)

## Data Availability

The data that support the findings of this study are available from the corresponding author upon reasonable request.
