# Supplementary material for: The associations between playing a musical instrument and grey matter in older adults at risk for dementia: a whole-brain VBM analysis
Source: GeroScience. 2025 Sep 3;48(3):3687–701. doi: 10.1007/s11357-025-01844-x (PMC13356170; doi:10.1007/s11357-025-01844-x)
Supplement: Supplementary file 1 — (DOCX 1,155 KB) [file 11357_2025_1844_MOESM1_ESM.docx]

**Supplementary document 1**. Music for Neuroplasticity Questionnaire

*Healthy Brain Ageing Program and Sydney Conservatorium of Music*

*We are interested to know whether you have any experience with music and your interest in participating in a new activity centered around music. Please answer every question to the best of your ability.*

**Section 1: Lifetime Instrument Playing Experience**

|  | **Questions** | **Answers** |
| --- | --- | --- |
| 1.0 | Have you ever learned to play an instrument? |  Yes  No *(If* ***“NO”*** *please go straight to* ***question 1.8****)* |
| 1.2 | At what age? | ..................... years |
| 1.3 | For how many years? | ..................... years |
| 1.4 | Which instrument? | ............................................................... |
| 1.5 | How many instruments did you play? |  One  Two  More than two |
| 1.6 | Do you continue to play/practice the music instrument? |  Yes  No (I*f* ***“NO”*** *please go straight to* ***question*** ***1.6.3****)* |
| 1.6.1 | How many hours per week do you play/practice the music instrument? | ..................... hours |
| 1.6.2 | Do you practice in private or in a group? |  Private  Group  Both  (P*lease go straight to* ***question 1.7****)* |
| 1.6.3 | ***If answered “NO” to question 1.6:***  When did you stop playing/ practicing? |  Less than a year ago   1 to 5 years ago   More than 5 years ago   More than 10 years ago |
| 1.6.4 | Why did you stop playing/ practicing? | .......................................................................................................... |
| 1.6.5 | How many hours per week did you use to play/practice the music instrument? | ….................. hours |
| 1.7 | Are you a professional musician? |  Yes  No |
| 1.8 | Are you able to read music? |  No  Yes A little  *(If* ***“YES”*** *please go straight to* ***Section 2****)* |
| 1.9 | Are you interested in learning how to read music alongside a musical instrument? |  Yes  No |

**Section 2: Lifetime Singing Experience**

|  | **Questions** | **Answers** |
| --- | --- | --- |
| 2.0 | Have you ever engaged in singing in an organised group (e.g. choir) and/or had vocal and singing lessons? |  Yes  No *(If* ***“NO”*** *please go straight to* ***Section 3****)* |
| 2.1 | At what age? | ..................... years |
| 2.2 | For how many years? | ..................... years |
| 2.3 | Do you continue to sing in an organised group or/and are you still taking singing lessons? |  Yes  No (I*f* ***“NO”*** *please go straight to question* ***2.3.2****)* |
| 2.3.1 | How many hours per week do you sing in an organise group or take signing lessons? | ..................... hours  (P*lease go straight to* ***Section 3****)* |
| 2.3.2 | ***If answered “NO” to question 2.3:***  When did you stop? |  Less than a year ago  1 to 5 years ago   More than 5 years ago  More than 10 years ago |
| 2.3.3 | Why did you stop? | .......................................................................................................... |
| 2.3.4 | How many hours per week did you use to sing in an organise group or take signing lessons? | ….................. hours |

**Section 3: Interest in learning music**

|  | **Questions** | **Answers** |
| --- | --- | --- |
| 3.0 | Would you consider taking up a new activity centered around music? |  No  Yes *(If* ***“YES”*** *please go straight to question* ***3.2****)* |
| 3.1 | Would the barrier be: | Time? Cost? Financial Lack of interest Lack of talent/skill for music  Other, please specify:………………….  **(P*lease go straight to the end of the questionnaire)*** |
| 3.2 | ***If answered “YES” to question 3.0:***  Would you like to learn music: |  Individually  In a group |
| 3.3 | Please rank the following instruments in order of your interest in learning each one (1 representing most interested; 5 representing least interested): | The Piano: …………  Guitar: …………  String instrument:……………  Wind instrument:……………  Other:………… please specify…………………………. |
| 3.4 | Would you attend a course/program at least once per week? | Yes  No *(If* ***“NO”*** *please go straight* ***to the end of the questionnaire)*** |
| 3.4.1. | What would be the main purpose of engaging in a music training program? |  To be connected to the arts   To learn a new skill   To meet new people   To promote brain neuroplasticity (i.e. growth of new brain cells) |
| 3.4.2 | Would you spend at least two hours practicing per week over a one year period? |  No Yes |

***Thank you for completing this questionnaire.***

| **Supplementary Table 1.** Demographic and clinical data for the three groups: Currently playing instruments (active players), history of playing instruments (former players) or those whom have never played a musical instrument (naïve) | | | | | | | | | | | | | | | | | | | | | | | | | |
| --- | --- | --- | --- | --- | --- | --- | --- | --- | --- | --- | --- | --- | --- | --- | --- | --- | --- | --- | --- | --- | --- | --- | --- | --- | --- |
| Measure | Active Players | | |  | | Former Players | | | | | | |  | | Naïve | | | | | | df | | | p-value |  |
|  | N | Mean (Md) | SD (IQR) |  | | | | N | Mean (Md) | | | SD (IQR) |  | | N | | | Mean (Md) | SD (IQR) | |  |  |  |  |  |
| Age (years) | 11 | 67.63 (70.25) | 8.75 | |  | | 20 | | | 69.10 (66.67) | 9.13 | | |  | | 26 | 70.61 (70.83) | | | 6.66 | | 56 | 0.567 | | |
| Sex: % female (n/N) | 11 | 36.36 | (4/11) | |  | | 20 | | | 70.00 | (14/20) | | |  | | 26 | 61.16 | | | (16/26) | | 2 | 0.182 | | |
| Education (years) | 11 | 15.45 (16.00) | 2.81 | |  | | 20 | | | 16.05 (16.00) | 2.28 | | |  | | 26 | 14.50 (15.00) | | | 3.00 | | 56 | 0.163 | | |
| MCI classification: % (n/N) | 11 | 45.45 | (5/11) | |  | | 20 | | | 35.00 | (7/20) | | |  | | 26 | 42.31 | | | (11/26) | | 2 | 0.819 | | |
| MCI subtypes: % amnestic MCI (n/N) | 5 | 80.00 | (4/5) | |  | | 7 | | | 100.00 | (7/7) | | |  | | 11 | 63.64 | | | (7/11) | | 2 | 0.167 | | |
| MCI domains: % multiple MCI (n/N) | 5 | 80.00 | (4/5) | |  | | 7 | | | 85.71 | (6/7) | | |  | | 11 | 72.72 | | | (8/11) | | 2 | 0.804 | | |
| Cognition (MMSE)* | 11 | 29.18 (29.00) | 0.96 (1.00) | |  | | 20 | | | 28.95 (29.00) | 1.39 (2.00) | | |  | | 25 | 28.56 (29.00) | | | 1.78 (3.00) | | 55 | 0.795 | | |
| Right-handedness: % (n/N) | 11 | 100.00 | (11/11) | |  | | 20 | | | 100.00 | (20/20) | | |  | | 26 | 88.46 | | | (23/26) | | 2 | 0.437 | | |
| Antidepressant use: % (n/N) | 11 | 27.27 | (3/11) | |  | | 19 | | | 20.00 | (4/20) | | |  | | 25 | 28.00 | | | (7/25) | | 2 | 0.861 | | |
| Depressive symptoms (GDS-15)* | 11 | 2.91 (2.00) | 4.39 (3.00) | |  | | 20 | | | 2.55 (2.00) | 2.63 (2.00) | | |  | | 26 | 3.46 (1.00) | | | 4.20 (8.25) | | 56 | 0.951 | | |
| Body Mass Index* | 11 | 26.18 (26.00) | 3.40 (6.16) | |  | | 18 | | | 25.87 (24.85) | 6.35 (6.33) | | |  | | 23 | 26.94 (24.70) | | | 5.17 (6.40) | | 51 | 0.659 | | |
| Medical burden (CIRS-G) | 11 | 6.91 (7.00) | 2.02 | |  | | 19 | | | 6.68 (6.00) | 4.77 | | |  | | 25 | 6.92 (7.00) | | | 2.89 | | 54 | 0.973 | | |
| Alcohol, units per week* | 10 | 4.40 (1.50) | 7.43 (6.50) | |  | | 18 | | | 4.03 (1.50) | 4.51 (8.25) | | |  | | 25 | 4.46 (3.00) | | | 4.47 (7.00) | | 52 | 0.807 | | |
| Age at which began learning music (years)^+^ | 11 | 13.27 (11.00) | 7.49 (12.00) | |  | | 20 | | | 9.20 (9.50) | 2.24 (2.75) | | |  | | - | - | | | - | | 30 | 0.138 | | |
| Years of music practice^+^ | 11 | 12.27 (6.00) | 14.57 (12.00) | |  | | 20 | | | 8.90 (7.50) | 3.65 (3.75) | | |  | | - | - | | | - | | 30 | 0.404 | | |
|  | | | | | | | | | | | | | | | | | | | | | | | | | |

Abbreviations: MCI = Mild cognitive impairment; MMSE, Mini-Mental State Examination; CIRS-G = Cumulative Illness Rating Scale-Geriatric; GDS-15 = 15-item Geriatric Depression Scale; SD = standard deviation; Md = Media; IQR = Interquartile range.

Note: All test statistics were one-way ANOVA analyses unless otherwise specified. Chi-square test was used for categorical data: antidepressant use, gender and MCI diagnosis, subtypes, and domains. All missing data were excluded using pairwise deletion. Bold values represent significant differences p < 0.05.

*Kruskal-Wallis H test.

^+^Mann-Whitney U test.

**Supplementary Table 2.** VBM sensitivity analysis

| Structure | Number of voxels | Peak MNI coordinate | | | p-value | T value |
| --- | --- | --- | --- | --- | --- | --- |
|  |  | x | y | z |  |  |
| Contrast 1* | | | | | | |
| Left Planum Temporale | 354 | -45 | -2 | -14 | **< 0.0001** | 5.20 |
| Left Planum Polare | 180 | -63 | -20 | 8 | **<0.0001** | 4.93 |
| Left precuneus | 449 | 2 | -70 | 48 | **<0.0001** | 4.81 |
| Right precuneus |  | -9 | -75 | 52 | **<0.0001** | 4.18 |
| Left Subcallosal Area | 146 | 4 | 21 | -16 | **<0.0001** | 4.83 |
| Right Subcallosal Area |  | -6 | 15 | 20 | **<0.0001** | 3.90 |
| Right Posterior Insula | 228 | 42 | 0 | -3 | **<0.0001** | 4.62 |
| Right inferior temporal gyrus | 77 | 50 | -34 | -28 | **<0.0001** | 4.74 |
| Contrast 2* | | | | | | |
| Left Cerebellum Exterior | 379 | -28 | -69 | -48 | **<0.0001** | 4.73 |
| Right precuneus | 252 | -2 | -69 | 48 | **<0.0001** | 4.68 |
| Left precuneus |  | 6 | -74 | 58 | **<0.0001** | 4.10 |

*Contrasts: Older adults who currently play a musical instrument compared to: (1) older adults without music experience; (2) older adults who have more than five years of music experience but stopped playing more than 10 years ago.

Analyses are reported at p < 0.0001, uncorrected for multiple comparisons with a minimum cluster size of 75 voxels. For this analysis, subjects who began learning music after the age of 50 were excluded.

*Bold values represent significant differences p < 0.0001.*

| **Supplementary Table 3.**  Associations between neuropsychological performance and music experience | | | | | | | | | | | | | | | | | | | | |  |
| --- | --- | --- | --- | --- | --- | --- | --- | --- | --- | --- | --- | --- | --- | --- | --- | --- | --- | --- | --- | --- | --- |
| Measure | | | | | Active Players | | | |  | Former Players | | |  | Naïve | | | Wald X^2^ | | η²ₚ | p-value | |
|  |  |  |  |  | N | Mean (Md) | | IQR |  | N | Mean (Md) | IQR |  | N | Mean (Md) | IQR |  |  |  |  |  |
| Verbal memory composite | | | | | 11 | | 0.26 (1.07) | 1.33 |  | 19 | 0.39 (0.65) | 1.73 |  | 26 | 0.24 (0.55) | 1.65 | 0.16 | | 0.003 | 0.925 | |
| Verbal learning composite | | | | | 11 | | 0.03 (0.71) | 1.26 |  | 19 | 0.53 (0.61) | 1.14 |  | 26 | 0.01 (0.08) | 1.58 | 3.11 | | 0.053 | 0.211 | |
| Executive functioning composite | | | | | 11 | | 0.67 (0.51) | 0.39 |  | 19 | 0.51 (0.49) | 0.70 |  | 26 | 0.28 (0.34) | 0.66 | 3.55 | | 0.061 | 0.169 | |
| Note: Active Player = Currently playing an instrument; Former Players = More than five years of music experience but stopped playing more than 10 years ago; Naïve = No instrument-playing experience; Md = Media; IQR = Interquartile range; η²ₚ = Partial Eta Squared.  All test statistics were General Linear Models, controlling for age.  *Bold values represent significant differences p < 0.05.* | | | | | | | | | | | | | | | | | | | | |  |
|  |  |  |  |  | | | | | | | | | | | | |  |  | |  |  |


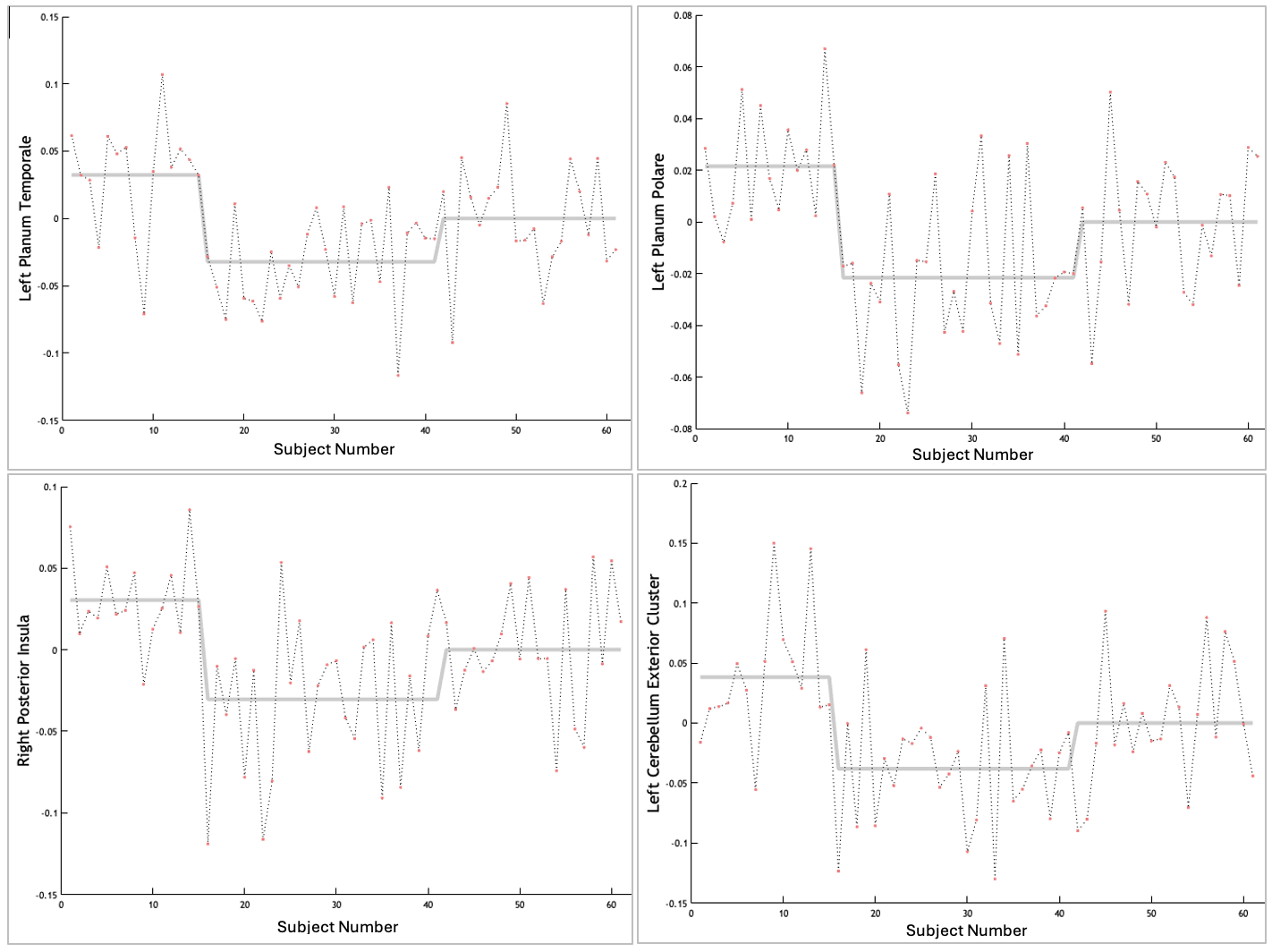


**Supplementary Figure 1.** Subject by subject fitted response of those who currently play a musical instrument (subject number 1 to 15), those without any music experience (subject number 16 to 41) and those with a history of music experience but stopped more than ten years ago (subject number 42 to 61) for the left planum temporale and planum polare, right posterior insula, and left cerebellum exterior (solid grey line = fitted response; red dots = dashed line connects subject-specific estimates).


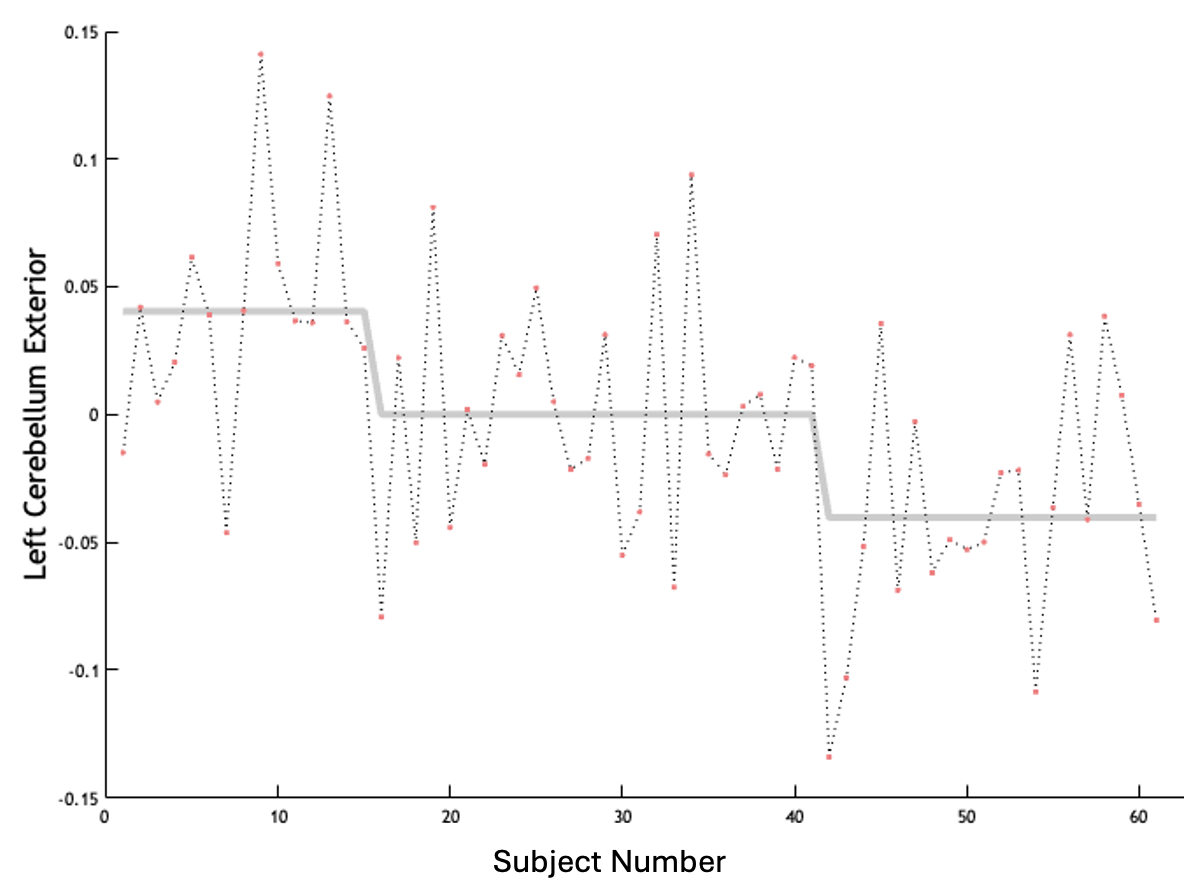


**Supplementary Figure 2.** Subject by subject fitted response of those who currently play a musical instrument (subject number 1 to 15), those without any music experience (subject number 16 to 41) and those who used to play music but stopped more than ten years ago (subject number 42 to 61) for the left cerebellum exterior (solid grey line = fitted response; red dots = dashed line connects subject-specific estimates).


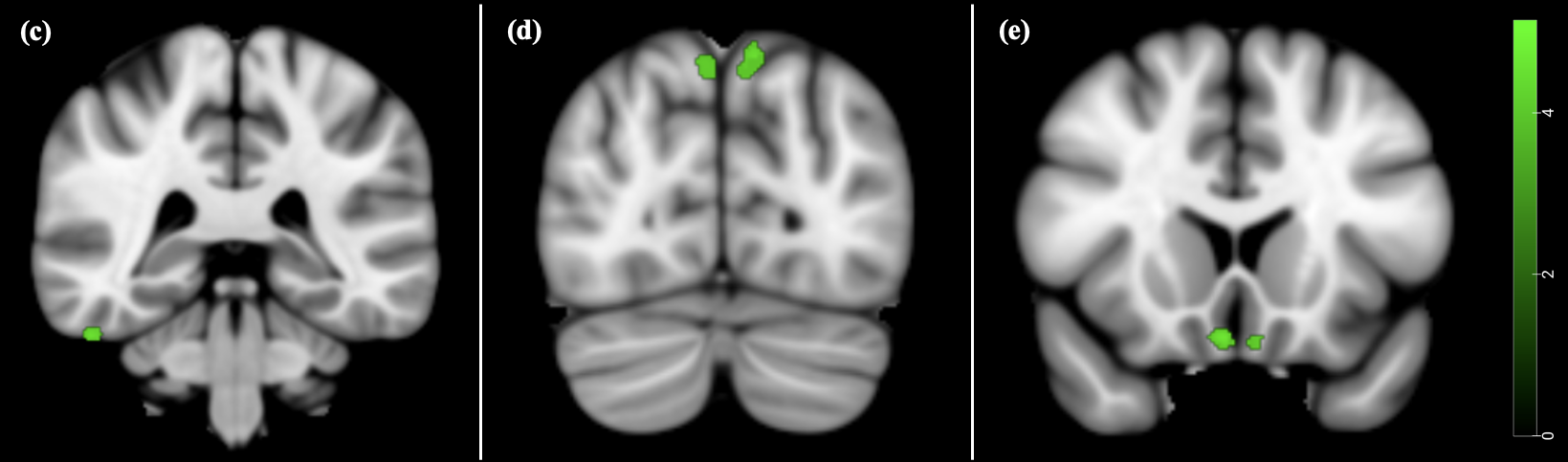


**Supplementary Figure 3. VBM sensitivity analysis: Additional brain regions showing grey matter density difference between those who currently play a musical instrument and those without music experience.** Compared to those without music experience, those who continued playing an instrument showed greater GM density in the (c) right inferior temporal gyrus, (d) bilateral precuneus, and (e) bilateral subcallosal area.

*Note: Age and TIV were included as covariates in the model. Analyses are reported at p < 0.0001, uncorrected for multiple comparisons with a minimum cluster size of 75 voxels. For this analysis, subjects who began learning music after the age of 50 were excluded. Colours represent t-values.*


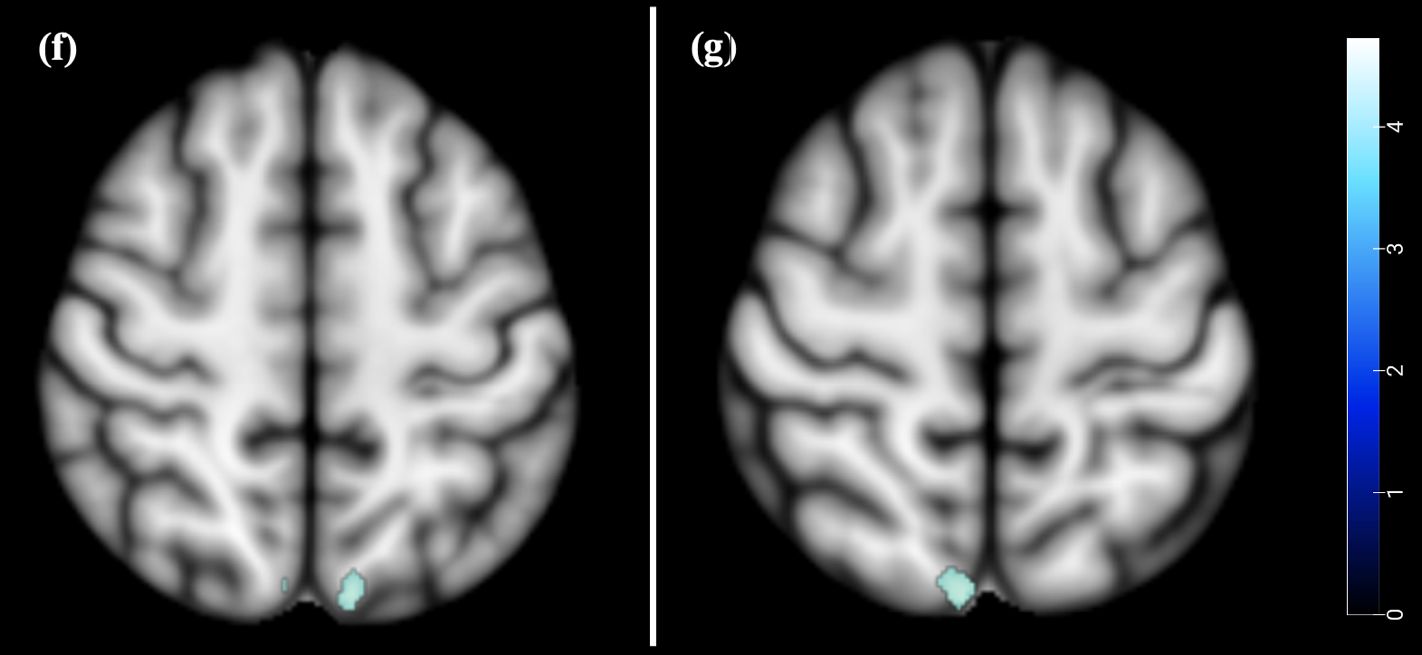


**Supplementary Figure 4. VBM sensitivity analysis: Additional brain regions showing grey matter density difference between those who currently play a musical instrument and those who used to play music but stopped more than ten years ago.** Compared to who used to play an instrument but stopped, those who continued playing an instrument showed greater GM density in the (f) left precuneus, and (g) right precuneus.

*Note: Age and TIV were included as covariates in the model. Analyses are reported at p < 0.0001, uncorrected for multiple comparisons with a minimum cluster size of 75 voxels. For this analysis, subjects who began learning music after the age of 50 were excluded. Colours represent t-values.*
